# Associations of Rap1 with Cell Wall Integrity, Biofilm Formation, and Virulence in Candida albicans

**DOI:** 10.1128/spectrum.03285-22

**Published:** 2022-11-23

**Authors:** Wen-Han Wang, Ting-Xiu Lai, Yi-Chia Wu, Zzu-Ting Chen, Kuo-Yun Tseng, Chung-Yu Lan

**Affiliations:** a Institute of Molecular and Cellular Biology, National Tsing Hua University, Hsinchu, Taiwan; b Department of Life Science, National Tsing Hua University, Hsinchu, Taiwan; c Taiwan Mycology Reference Center, National Institute of Infectious Diseases and Vaccinology, National Health Research Institutes, Zhunan Township, Miaoli County, Taiwan; d School of Medicine, National Tsing Hua University, Hsinchu, Taiwan; University of Guelph

**Keywords:** *Candida albicans*, Rap1, cell wall integrity, biofilm, pathogenesis, virulence, biofilms

## Abstract

Rap1 (repressor activator protein 1) is a multifunctional protein, playing important roles in telomeric and nontelomeric functions in many eukaryotes. Candida albicans Rap1 has been previously shown to be involved in telomeric regulation, but its other functions are still mostly unknown. In this study, we found that the deletion of the *RAP1* gene altered cell wall properties, composition, and gene expression. In addition, deletion of *RAP1* affected C. albicans biofilm formation and modulated phagocytosis and cytokine release by host immune cells. Finally, the *RAP1* gene deletion mutant showed attenuation of C. albicans virulence in a Galleria mellonella infection model. Therefore, these findings provide new insights into Rap1 functions that are particularly relevant to pathogenesis and virulence of C. albicans.

**IMPORTANCE** C. albicans is an important fungal pathogen of humans. The cell wall is the outermost layer of C. albicans and is important for commensalism and infection by this pathogen. Moreover, the cell wall is also an important target for antifungals. Studies of how C. albicans maintains its cell wall integrity are critical for a better understanding of fungal pathogenesis and virulence. This work focuses on exploring unknown functions of C. albicans Rap1 and reveals its contribution to cell wall integrity, biofilm formation, and virulence. Notably, these findings will also improve our general understanding of complex machinery to control pathogenesis and virulence of fungal pathogens.

## INTRODUCTION

Candida albicans is a commensal yeast inhabiting multiple body sites of healthy individuals, normally being innocuous (1). However, changes in the internal environment of the host, as exemplified by that in immunocompromised individuals, can promote C. albicans to become pathogenic and cause various infections including life-threatening disseminated candidiasis (2, 3). Therefore, the ability of C. albicans to adapt to changing environmental conditions is critical for its survival and pathogenicity.

The cell wall is the outermost structure of C. albicans, playing a vital role in maintenance of cell integrity, interaction with the host environment, and fungal pathogenesis (4, 5). Moreover, the cell wall is also one of the main targets of antifungal drugs (6, 7). The C. albicans cell wall is composed mainly of proteins and polysaccharides, forming two layers visualized by electron microscopy (8). The inner layer includes β-1,3-glucan, β-1,6-glucan, and chitin to form the core skeletal structure of the wall (8, 9). Moreover, the outer layer of the cell wall is enriched with heavily glycosylated mannoproteins cross-linked to β-1,3-glucan (10) and generally masks the inner β-glucan layer to reduce recognition of C. albicans by the host immune system (11, 12).

Although the C. albicans cell wall is tough, it can be flexible to change the relative amounts of its composition in response to the environment (13–15). This underlying composition remodeling is thus crucial for the maintenance of cell wall integrity (CWI) and is regulated by various signaling pathways, including the Mkc1, Hog1, and Cek1 mitogen-activated protein (MAP) kinase cascades (15, 16). Moreover, a variety of transcription factors also contribute to the regulation of C. albicans CWI in response to external stresses. For example, Cas5, Czf1, and Rlm1 are on the list of CWI regulators (17–19). Together, maintaining CWI requires complex interplays among multiple signaling pathways and transcription regulators.

Repressor activator protein 1 (Rap1) is a conserved DNA-binding protein identified in yeasts, protozoa, and mammalian cells (20). In the yeast Saccharomyces cerevisiae, Rap1 (ScRap1) plays an important role in telomere regulation by directly binding to double-stranded telomeric DNA. The molecular events regulated by ScRap1 are exemplified by subtelomeric gene silencing (21), telomere length control (22), and telomere end protection (23–25). Nevertheless, ScRap1 is also involved in other functions not relevant to telomere regulation, including transcriptional regulation, metabolic control, and oxidative stress response (26, 27). In C. albicans, a homolog of ScRap1 has been identified and characterized. Similarly, C. albicans Rap1 participates in the control of telomere length and structure (28–30). However, nontelomeric functions of C. albicans Rap1 are still largely unknown.

In this study, we have begun to reveal other functions of C. albicans Rap1, particularly those related to C. albicans pathogenesis. We demonstrated that deletion of C. albicans *RAP1* alters cell wall properties, composition, and expression of genes related to cell wall biosynthesis and remodeling. Moreover, the deletion of *RAP1* promoted activation of Mkc1 and Cek1 kinases and impacted C. albicans biofilm formation. Finally, the *RAP1* gene deletion (*rap1*Δ/Δ) mutant also affected C. albicans-macrophage interaction and exhibited attenuated virulence in a Galleria mellonella infection model.

## RESULTS

### Deletion of *RAP1* impacts C. albicans cell wall properties.

**FIG 1:**
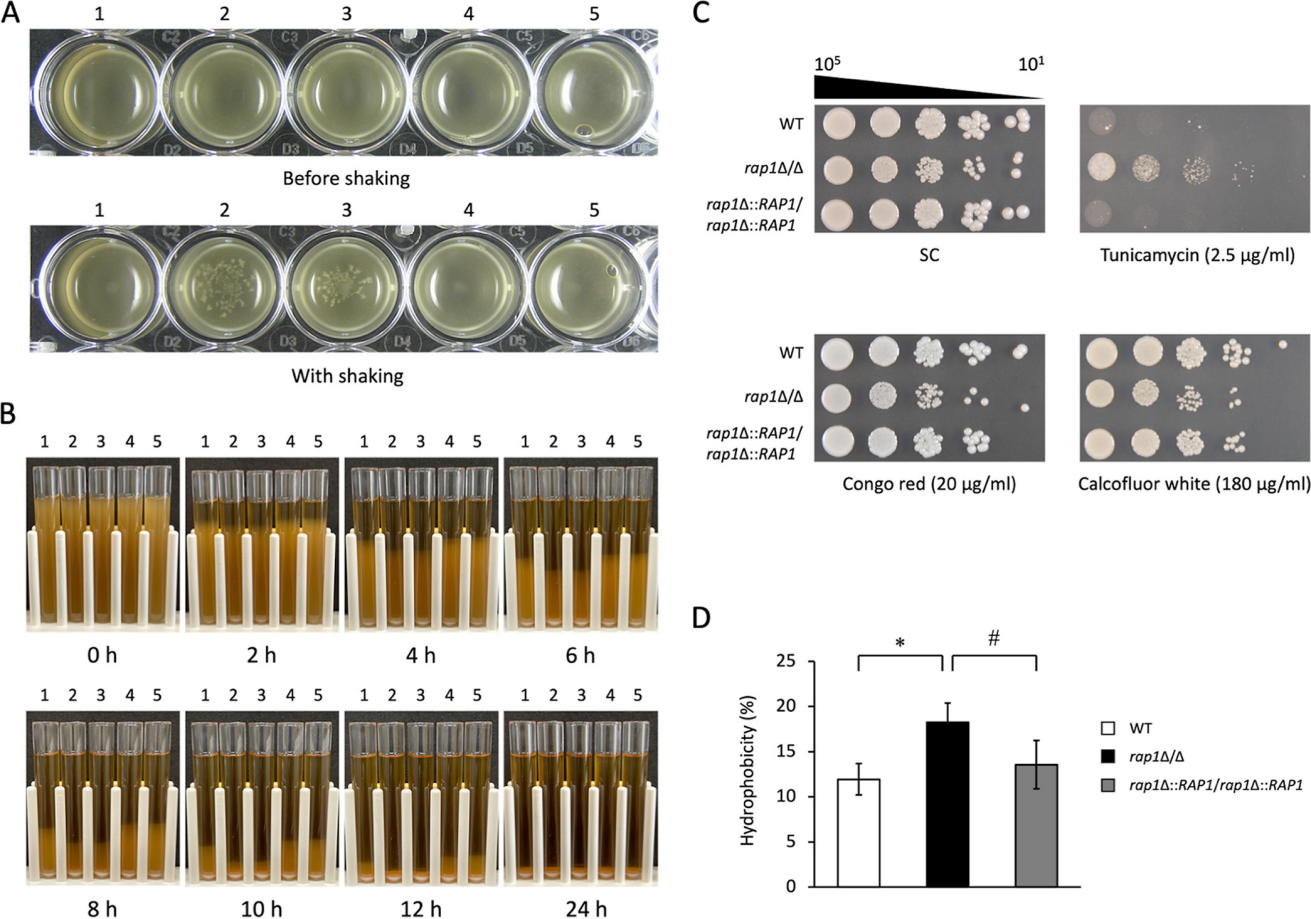
The *RAP1* deletion causes alterations in cell wall-related properties in C. albicans. (A) Cell aggregation. Overnight cultures were transferred to each well of 24-well microplates and photographed immediately (top panel). Then, cells were shaken slowly (100 rpm) at 25°C for 30 min and photographed (bottom panel). A representative image from three independent experiments with identical results is shown. 1, wild type; 2 and 3, the *rap1*Δ/Δ mutants; 4 and 5, the *RAP1*-reintegrated strains. (B) Cell sedimentation. Overnight cultures were sedimented by standing at room temperature and photographed at different periods of time as indicated. (C) Cell susceptibility to cell wall-perturbing agents. Overnight cultures were spotted onto SC with or without drugs as indicated. Cells were incubated at 30°C for 3 days. A representative image from three independent experiments with identical results is shown. WT, wild type. (D) CSH assay. Cells were grown overnight, and CSH was determined. The results are displayed as the mean ± standard deviation from three independent experiments. *, *P* < 0.05; #, *P* = 0.076.

To explore nontelomeric functions of C. albicans Rap1, we generated the *rap1*Δ/Δ mutant and *RAP1*-reintegrated strains. The successful strain construction was verified by PCR analysis of genomic DNA (see Fig. S1 in the supplemental material). Moreover, the *rap1*Δ/Δ mutant was viable, indicating C. albicans *RAP1* is not essential, which is consistent with previous findings (28–30). Interestingly, we further found that the *rap1*Δ/Δ mutant, but not the wild type and *RAP1*-reintegrated controls, aggregated and formed flocs during cell growth in microplates (Fig. 1A). The formation of flocs was also assayed by sedimentation rate, showing that the *rap1*Δ/Δ mutant sediments faster than the controls (Fig. 1B).

Since flocculation and sedimentation are known to be cell wall associated (31, 32), we thus hypothesized that deletion of *RAP1* may lead to changes in the properties of the C. albicans cell wall. To test this hypothesis, cell susceptibility to cell wall-perturbing drugs was first examined. As shown in Fig. 1C, the *rap1*Δ/Δ mutant differed from the controls in its susceptibility to cell wall-perturbing drugs. The *rap1*Δ/Δ mutant was more sensitive to calcofluor white and Congo red but more resistant to tunicamycin. Moreover, because cell surface hydrophobicity (CSH) contributes to C. albicans aggregation and adhesion (33), the CSH levels were also compared between the *rap1*Δ/Δ mutant and the wild-type and *RAP1*-reintegrated strains. Indeed, the CSH of the *rap1*Δ/Δ mutant was increased compared to the controls (Fig. 1D).

### Deletion of *RAP1* causes alterations of cell wall carbohydrate content.

**FIG 2:**
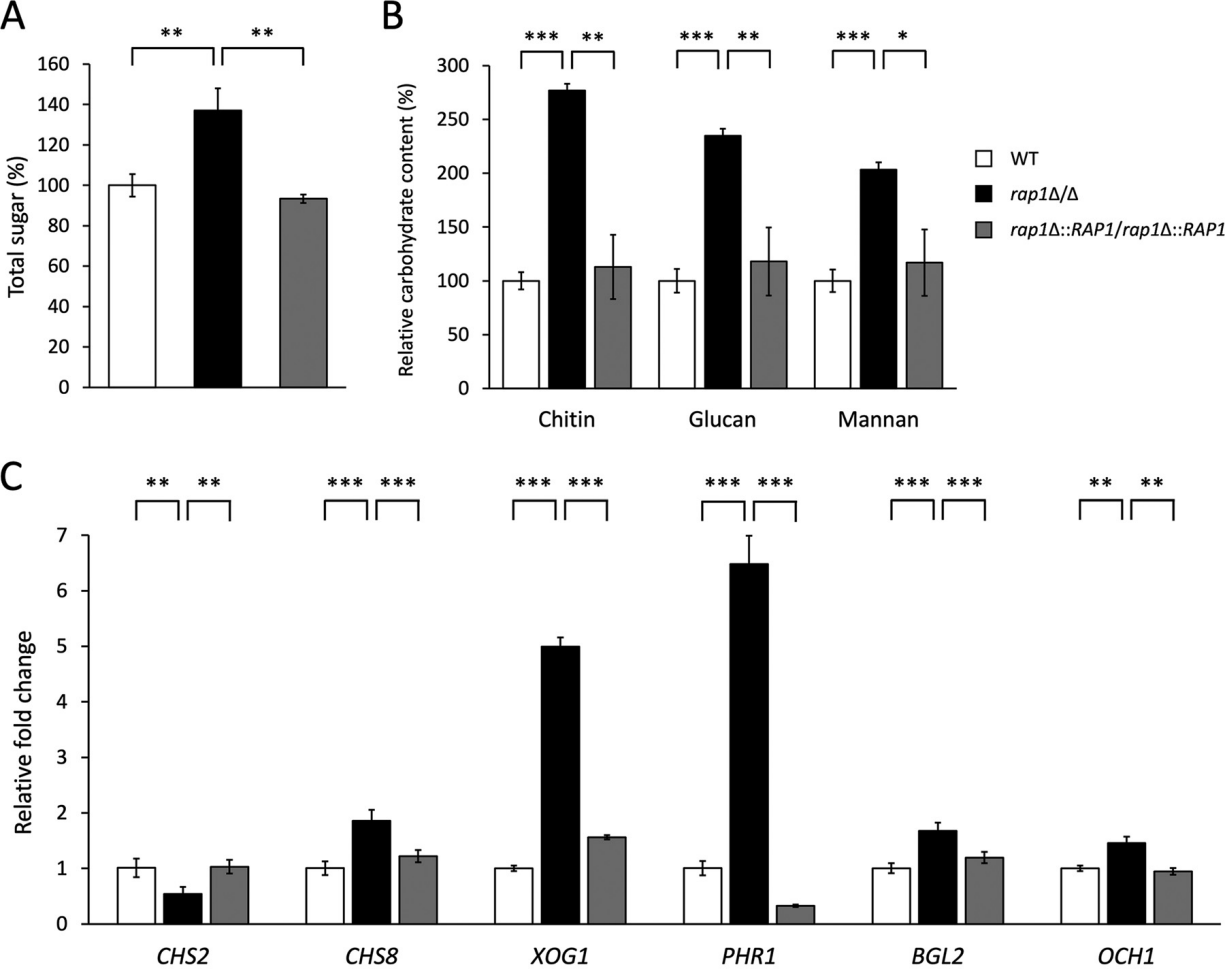
The *RAP1* deletion leads to changes in cell wall carbohydrate contents and the expression of cell wall-related genes. (A) Measurement of total carbohydrate contents of the cell wall. The results are displayed as the mean ± standard deviation from three independent experiments. **, *P* < 0.01. (B) Quantification of the cell wall chitin, glucan, and mannan content by HPAEC-PAD. The results are displayed as the mean ± standard deviation from three independent experiments. *, *P* < 0.05; **, *P* < 0.01; ***, *P* < 0.001. WT, wild type. (C) The expression of cell wall biosynthesis and remodeling genes. RT real-time qPCR was performed, and *ACT1* transcripts were used as an internal control. The results are presented as the mean ± standard deviation from at least three independent experiments. **, *P* < 0.01; ***, *P* < 0.001.

The cell wall consists of polysaccharides and proteins, and the former account for a large portion of the dry weight of the cell wall (5). Moreover, CSH is closely associated with cell wall compositions (34). Therefore, carbohydrate content in the cell wall was also measured. Total carbohydrate content in the cell wall was increased by approximately 40% in the *rap1*Δ/Δ mutant compared to the controls (Fig. 2A). Glucan, mannan, and chitin are the three major polysaccharides of the C. albicans cell wall. To further examine the effect of *RAP1* deletion, the content of each polysaccharide was quantified using high-performance anion-exchange chromatography with pulsed amperometric detection (HPAEC-PAD) analysis. The results show that contents of all three polysaccharides were increased in the *rap1*Δ/Δ mutant (Fig. 2B).

Because deletion of *RAP1* affects cell wall properties and composition, we suspected that Rap1 may act as a transcription regulator to control the expression of cell wall-related genes. To test this possibility, reverse transcription (RT) real-time quantitative PCR (qPCR) analysis was performed to measure expression of genes encoding enzymes for cell wall biosynthesis and remodeling (35, 36). Although there was no significant difference in *CHS1*, *CHS3*, *FKS1*, and *FKS2* gene expression (Fig. S2 in the supplemental material), modulation of many other genes was detected in the *rap1*Δ/Δ mutant compared to the wild-type and *RAP1*-reintegrated strains (Fig. 2C). Among these differentially expressed genes, *CHS2* and *CHS8* are involved in chitin synthesis. Moreover, increased expression was shown in the *XOG1*, *PHR1*, and *BGL2* genes (~5-, 6.5-, and 1.7-fold), which encode exo-1,3-β-glucanase, glycosidase, and 1,3-β-glucosyltransferase, respectively (37–39). Finally, the *OCH1* gene product is a mannosyltransferase, participating in mannosylation of cell wall proteins (2, 10).

### The Mkc1 and Cek1 MAP kinases are activated in the *rap1*Δ/Δ mutant.

In response to environmental changes and stresses, distinct signaling pathways are activated in C. albicans. Importantly, the effect of many environmental factors is transduced through three major MAP kinase pathways in C. albicans, including the Mkc1, Cek1, and Hog1 cascades (40–42). The Mkc1 pathway is associated with the maintenance of CWI mainly due to its functions in cell wall biogenesis (43–45). Moreover, the Cek1 and Hog1 pathways are also known to contribute to cell wall construction (46–48).

**FIG 3:**
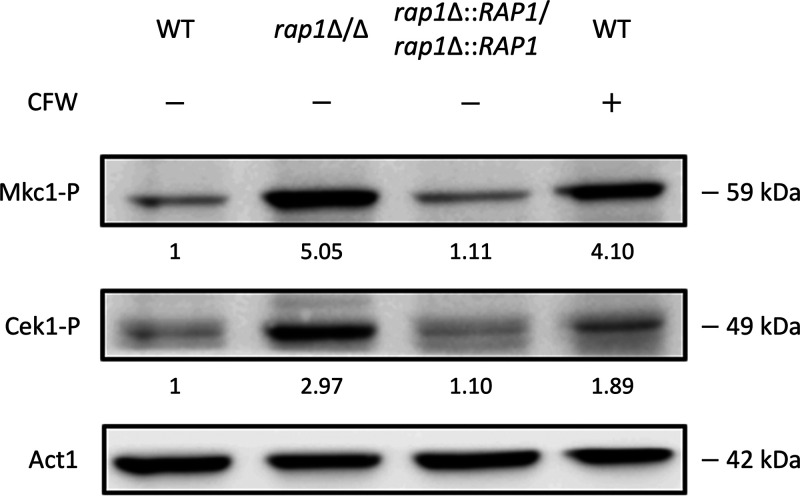
Mkc1 and Cek1 are activated in the *rap1*Δ/Δ mutant. Mkc1 and Cek1 activation were detected by Western blot analysis. Equal amounts of proteins (25 μg) from each strain were loaded. Cells treated with calcofluor white (CFW) were used as a positive control. The phosphorylated Mkc1 (Mkc1-P) and Cek1 (Cek1-P) were analyzed using ImageJ software. Act1 was used as a loading control and was used to normalize Mkc1-P and Cek1-P levels indicated by the fold change values. The data are representative of three independent experiments with identical results.

Here, to further correlate Rap1 with CWI maintenance, we also determined activation of MAP kinases in the *rap1*Δ/Δ mutant using Western blot analysis. As shown in Fig. 3, the phosphorylation levels of Mkc1 and Cek1 were increased in the *rap1*Δ/Δ mutant compared to the wild-type and *RAP1*-reintegrated strains. However, there was no significant difference in the levels of phospho-Hog1 among all the strains examined (Fig. S3). Together, these results and the findings that *RAP1* deletion affects susceptibility to cell wall-perturbing drugs, cell wall properties and composition, and cell wall-related gene expression (Fig. 1 and 2) suggest the potential relationship between Rap1 and CWI in C. albicans.

### *RAP1* deletion also affects biofilm formation.

**FIG 4:**
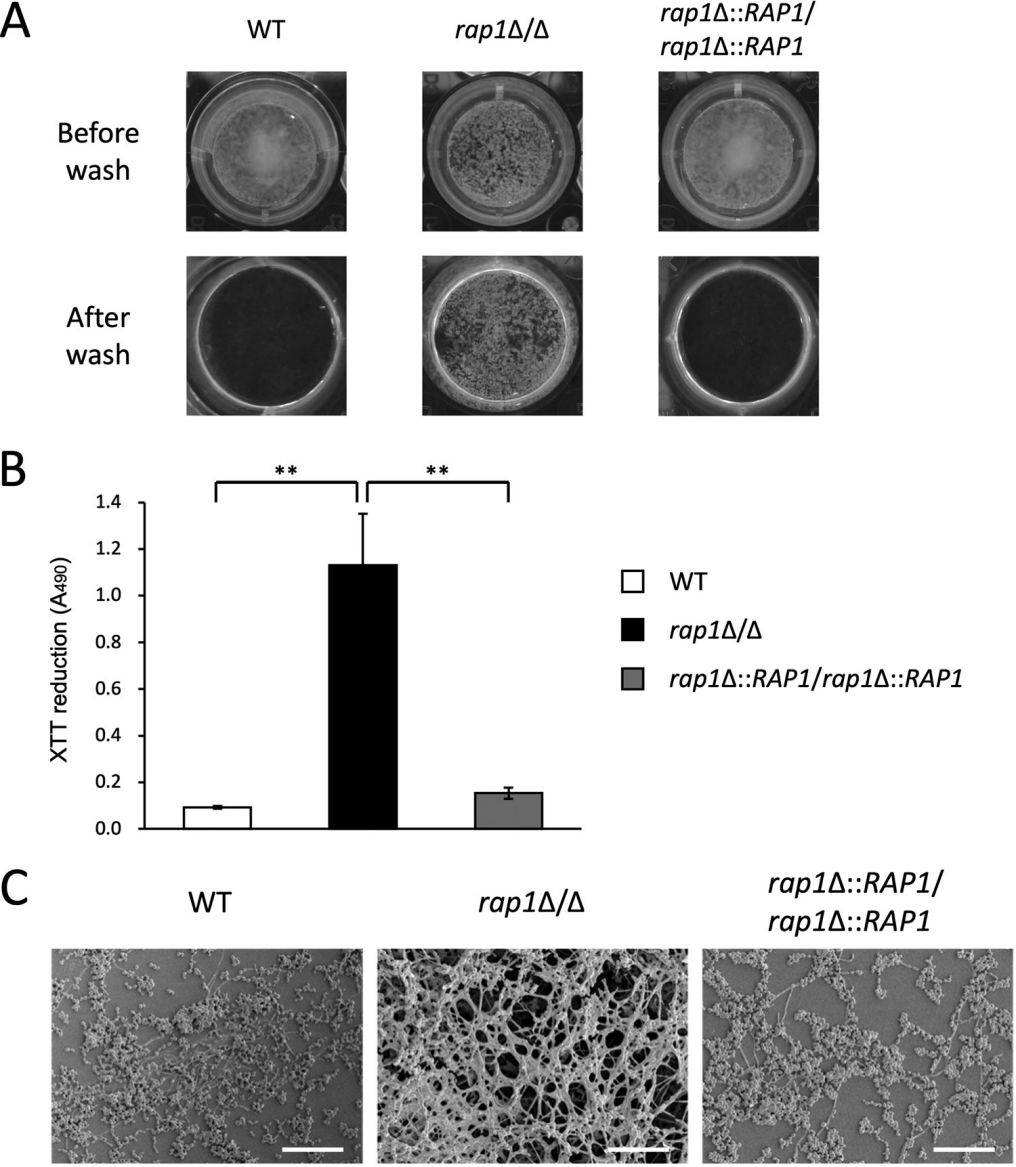
Rap1 is involved in biofilm formation. (A) Biofilms were formed in each well of a 24-well polystyrene microplate in SC medium and incubated at 37°C with 5% CO_2_ for 24 h. (B) Measurement of biofilm formation using the XTT reduction method. The results are presented as the mean ± standard deviation from three independent assays. **, *P* < 0.01. (C) Biofilm structure was examined using SEM. The cells were grown on polystyrene coverslips for 24 h to form biofilms. Pictures were taken at an ×1,000 magnification. Bars, 50 μm.

The cell wall is closely connected with biofilm formation on abiotic or biotic surfaces, which is important for pathogenicity of C. albicans (2, 14, 49). In addition, using a computational approach, Rap1 was identified as a transcription factor potentially related to C. albicans biofilm formation (50). Because of the influence of *RAP1* deletion on CWI (Fig. 1 to 3), we hypothesized that C. albicans biofilm formation might also be affected in the *rap1*Δ/Δ mutant. To assay the formation of biofilm, the XTT [2,3-bis-(2-methoxy-4-nitro-5-sulfophenyl)-2H-tetrazolium-5-carboxanilide] reduction method was used. Indeed, the *rap1*Δ/Δ mutant formed a robust biofilm compared to those of the wild-type and *RAP1*-reintegrated strains (Fig. 4A and B). Moreover, the structure of biofilm was also examined by scanning electron microscopy (SEM). As demonstrated in Fig. 4C, the wild-type and *RAP1*-reintegrated strains formed biofilms with a single layer of cells. However, the *rap1*Δ/Δ mutant formed a biofilm with a complicated three-dimensional structure (Fig. 4C). Concisely, *RAP1* deletion enhances C. albicans biofilm formation.

### Cell wall changes by *RAP1* deletion have impacts on C. albicans-macrophage interaction.

**FIG 5:**
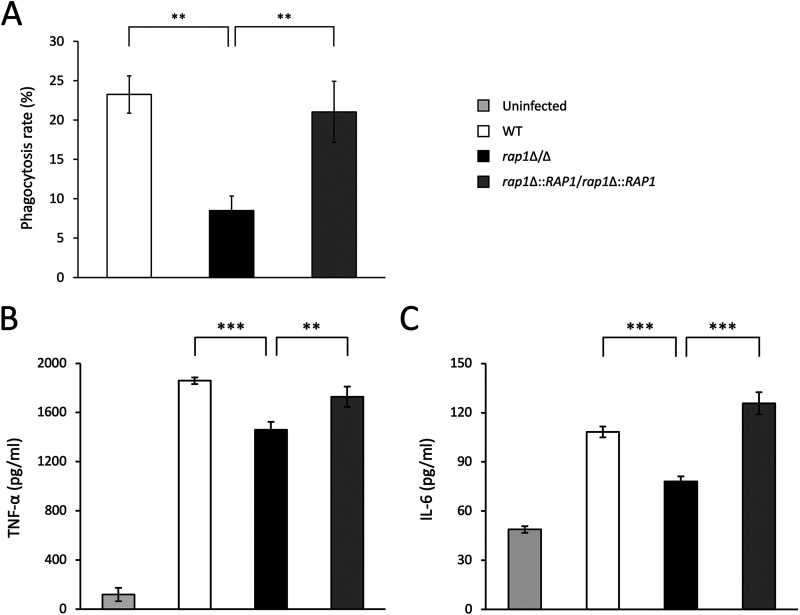
The *RAP1* deletion has impacts on C. albicans-macrophage interaction. (A) Macrophage phagocytosis assay. C. albicans cells were coincubated with J774A.1 macrophages (at an MOI of 3) for 30 min, and the phagocytosis rates were determined. The results are expressed as the mean ± standard deviation from three independent assays. **, *P* < 0.01. (B and C) Measurement of TNF-α (B) and IL-6 (C) production. C. albicans cells were coincubated with J774A.1 cells (at an MOI of 3), and production of cytokines was measured using ELISA. Uninfected J774A.1 cells were used as a negative control. The results are expressed as the mean ± standard deviation from three independent assays. **, *P* < 0.01; ***, *P* < 0.001.

Because the cell wall plays a critical role in the interaction of C. albicans with the host immune system, we reasoned that cell wall changes caused by *RAP1* deletion would affect immune recognition and response (51, 52). The rates of C. albicans engulfment and cytokine secretion by J774A.1 macrophages were thus examined. As indicated in Fig. 5A, a much lower percentage of cell uptake by macrophages was found in the *rap1*Δ/Δ mutant than in the wild-type and *RAP1*-reintegrated strains. Besides, macrophage-mediated responses were also determined by detecting cytokine secretion using an enzyme-linked immunosorbent assay (ELISA). When macrophages were infected with the *rap1*Δ/Δ mutant, the levels of tumor necrosis factor alpha (TNF-α) and interleukin-6 (IL-6) secretion were lower than those in macrophages infected with the wild-type and *RAP1*-reintegrated strains (Fig. 5B and C). These results suggest that *RAP1* deletion-mediated cell wall changes give rise to diminished recognition and engulfment by macrophages and a reduced proinflammatory cytokine response.

### *RAP1* deletion attenuates C. albicans virulence.

**FIG 6:**
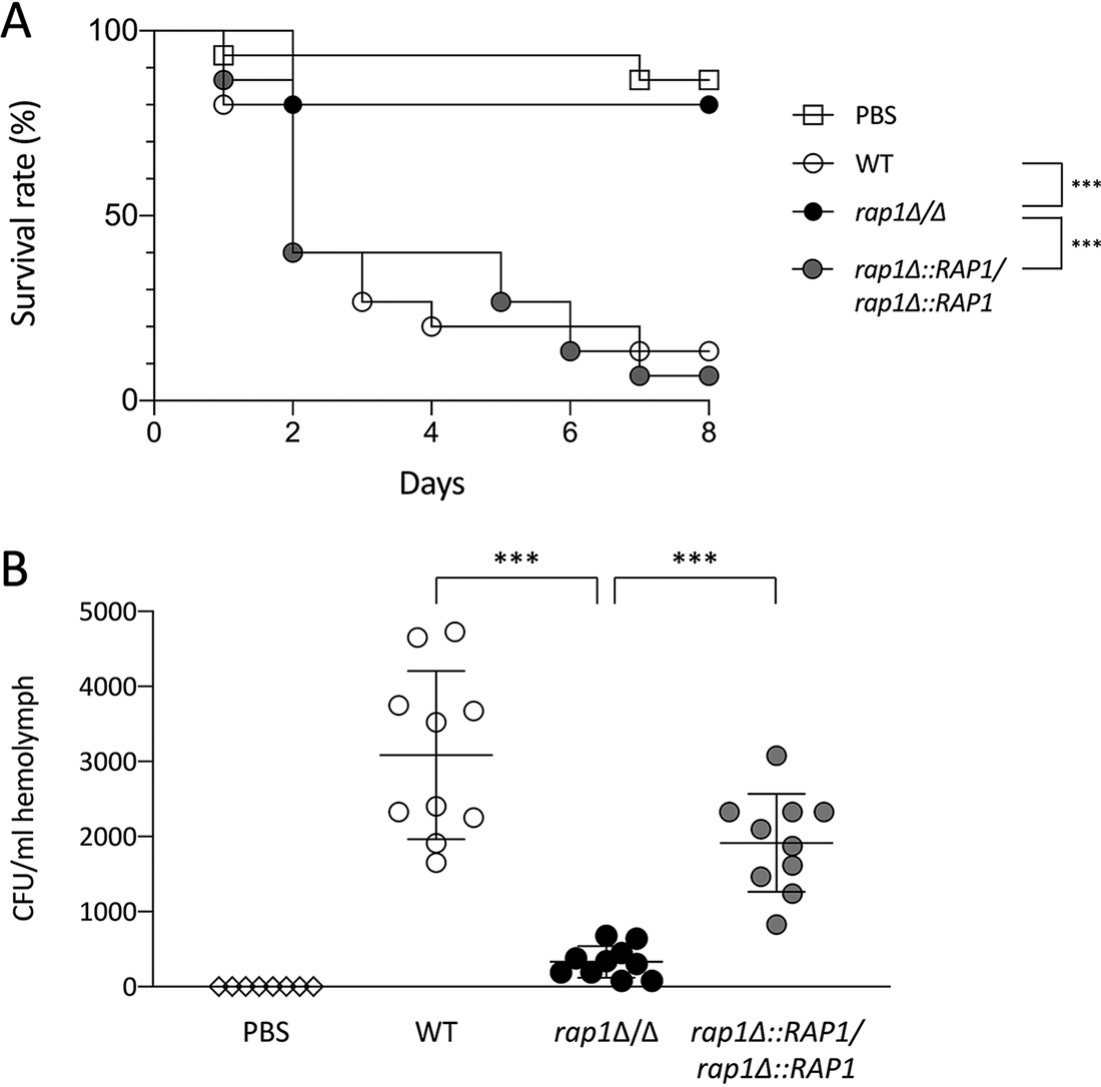
Rap1 contributes to C. albicans virulence. (A) Assessment of survival rate of G. mellonella. A total of 5 × 10^5^ C. albicans cells was injected into larvae of G. mellonella (*n* = 15 per C. albicans strain). The survival rates were monitored daily for 10 days. Larvae inoculated with PBS were used as a vehicle control. ***, *P* < 0.001. (B) Assessment of fungal load. A total of 6 × 10^5^ C. albicans cells was injected into larvae of G. mellonella (*n* = 10 per C. albicans strain). Hemolymph of larvae was collected 1 day postinfection and plated on YPD agar plates, and numbers of CFU were counted. Larvae inoculated with PBS were used as a control. Each symbol represents an individual larva. Horizontal lines show the mean ± standard deviation. ***, *P* < 0.001.

To investigate the influence of Rap1 on C. albicans virulence, a G. mellonella infection model was used. G. mellonella larvae infected with the *rap1*Δ/Δ mutant showed an overall higher survival rate than that of larvae infected with the wild-type and *RAP1*-reintegrated strains (Fig. 6A). Moreover, the fungal load from larvae infected with the *rap1*Δ/Δ mutant was significantly decreased compared to the load from those infected with the control strains (Fig. 6B). These results indicate that *RAP1* deletion attenuates C. albicans virulence.

## DISCUSSION

Rap1 is a conserved multifunctional protein involved in various cellular processes, of which telomere regulation is one of the most studied events (26, 27). For example, ScRap1 controls subtelomeric gene silencing (21) and telomere length (22) and protects chromosomes from DNA damage caused by unwanted DNA repair mechanisms and abnormal chromosome fusions (23–25, 53, 54). Although ScRap1 and its orthologs have similar functions in telomere regulation, interestingly, they can act differently. ScRap1 can directly recognize telomeric DNA, whereas human Rap1 (hRap1) cannot bind DNA directly and is recruited to telomeres through its protein partner hTRF2 (53). Moreover, ScRap1 and hRap1 play a similar role in other nontelomeric functions, including transcriptional control, metabolism, and oxidative stress response (26), while hRap1 contributes to inflammation related to human diseases (26, 55).

Apart from telomere regulation, both ScRap1 and hRap1 also act as transcription regulators. For example, ScRap1 activates genes encoding glycolytic enzymes and ribosomal proteins (56, 57). To study ScRap1 in transcription regulation, extensive studies have established structure-function relationships of the protein. ScRap1 can be roughly divided into three regions: the N-terminal and C-terminal domains and a central DNA-binding domain (DBD). The N-terminal domain is suggested to elicit DNA bending and contains a BRCT (BRCA1 C terminus) domain that interacts with ScGcr1 to regulate glycolytic genes (58, 59). The DBD of ScRap1 has two Myb motifs and can directly interact with the TATA-binding protein (54, 60). Finally, the C-terminal domain, working with other proteins such as ScRif1, ScRif2, ScSir3, and ScSir4, is required for regional silencing at several loci on chromatins (61–66).

In C. albicans, a homolog of ScRap1 was identified and characterized (28). Like ScRap1, C. albicans Rap1 was found to participate in controlling telomere length and structure (30). Intriguingly, there are several differences between ScRap1 and C. albicans Rap1. First, ScRap1 is an essential protein, whereas the C. albicans Rap1 is nonpivotal for cell viability (28). Second, C. albicans Rap1 lacks the C-terminal domain of ScRap1 (28–30). Finally, C. albicans Rap1 represses the formation of pseudohyphae under conditions favoring growth as the yeast form (28). The role of ScRap1 in pseudohyphal growth has not been reported in S. cerevisiae. Therefore, it would be interesting to further reveal similarities and differences between functions of ScRap1 and C. albicans Rap1.

This work aimed to explore previously unknown functions of C. albicans Rap1. Since *RAP1* is not essential in C. albicans, we generated the *rap1*Δ/Δ mutant to examine C. albicans *RAP1* gene function. Our results indicate that C. albicans Rap1 is closely associated with CWI. The *rap1*Δ/Δ mutant aggregated, formed flocs, and sedimented faster than the control strains (Fig. 1A and B). In addition, the deletion of *RAP1* caused alterations in cell surface hydrophobicity, cell susceptibility to cell wall-disrupting agents, and the chemical composition of the C. albicans cell wall (Fig. 1C and D and Fig. 2A and B). Interestingly, the N-terminal deletion mutant of ScRap1 was hypersensitive to cell wall-disrupting agents and changed the cell wall composition (67). These results suggest that the N-terminal domain of Rap1 may be also important in regulating CWI in C. albicans. However, further investigation is required. Finally, several cell wall biosynthesis and remodeling genes were modulated, and the Mkc1 and Cek1 MAP kinases were activated in the C. albicans *rap1*Δ/Δ mutant (Fig. 2C and Fig. 3). Together, our observations in the *rap1*Δ/Δ mutant provide evidence for the role of C. albicans Rap1 in regulating CWI.

Cell wall components, e.g., Als1, and CSH have been linked to C. albicans adhesion and biofilm formation (68, 69). According to our findings in Fig. 1D and Fig. 2A and B, the role of Rap1 in biofilm formation was thus determined. Biofilm formation in C. albicans involves complex processes including cell adhesion, yeast and hypha morphogenesis, and extracellular matrix (ECM) accumulation and dispersal (70, 71). Hyphae act as a supporting scaffold for yeast and other hyphal cells and contribute significantly to the overall architectural stability of the biofilm (71). Although deletion of *RAP1* affected C. albicans biofilm formation (Fig. 4A to C), intriguingly, we found that the *rap1*Δ/Δ mutant still retains the ability to form hyphae, similar to the control strains (see Fig. S4 in the supplemental material). Therefore, to examine whether deletion of *RAP1* has an impact on cell adhesion, ECM synthesis, and biofilm dispersal is of interest for future research.

Of note, components of the C. albicans cell wall represent the major pathogen-associated molecular patterns (PAMPs) that can be recognized by diverse pattern recognition receptors (PRRs) to trigger host immune responses (51, 72). Moreover, the cell wall is also important during C. albicans commensalism and infections (4, 11). Since cell wall properties and composition were changed in the *rap1*Δ/Δ mutant, we reasoned that *RAP1* deletion may also affect the pathogenesis and virulence of C. albicans. Thus, phagocytosis and cytokine release from murine macrophages against C. albicans were thus examined. Our results indicate the *rap1*Δ/Δ mutant is more resistant to being phagocytosed than the wild-type and *RAP1*-reintegrated strains (Fig. 5A). Moreover, lower levels of TNF-α and IL-6 were also detected from macrophages infected with the *rap1*Δ/Δ mutant than from macrophages infected with the control strains (Fig. 5B and C). Finally, G. mellonella larvae were used to assess the effects of *RAP1* deletion on C. albicans virulence. Indeed, a higher survival rate and a lower fungal burden were revealed from larvae infected with the *rap1*Δ/Δ mutant (Fig. 6A and B). In aggregate, our findings clearly show that Rap1 is associated with CWI, biofilm, and virulence in C. albicans.

In this work, we expanded the new roles of Rap1, particularly in the pathogenesis and virulence of C. albicans. However, many questions still need to be addressed. For example, it is still not clear whether C. albicans Rap1 directly or indirectly regulates target gene expression. In S. cerevisiae, the C-terminal domain of ScRap1 mediates the association of ScRap1 with other regulators to control transcription silencing of subtelomeric regions (73–75). Because C. albicans Rap1 lacks the C-terminal domain of ScRap1, it will be interesting to further examine the domain structure-function relationship of C. albicans Rap1. Moreover, ScRap1 has been found to target ~5% of yeast genes and contribute to activation of ~37% of RNA polymerase II-mediated transcription (76). Therefore, the consensus of C. albicans Rap1-regulated promoters is also required to be determined. Moreover, other signaling components (e.g., the calcium-calcineurin pathway) and transcription factors (e.g., Cas5, Czf1, Rlm1, and Bcr1) can also regulate CWI and biofilm formation in C. albicans (17–19, 77). Moreover, many other transcription factors have a role in biofilm formation (70). Further investigation will help us to understand the comprehensive cell wall regulating network.

## MATERIALS AND METHODS

### C. albicans strains, media, and growth conditions.

The C. albicans strains used in this work are listed in Table S1 (in the supplemental material). Cells were routinely maintained at −80°C and plated on YPD agar (1% yeast extract, 2% peptone, 2% glucose, and 1.5% agar) before each experiment. A single colony was inoculated into YPD broth and grown at 30°C overnight (~16 h) with shaking (180 rpm). The overnight culture was subcultured in synthetic complete (SC) medium (0.67% yeast nitrogen base [YNB] with ammonium sulfate, 0.079% complete supplement mixture [MP Biomedicals, Santa Ana, CA, USA], and 2% glucose) and grown at 30°C with shaking to the exponential phase. For the induction of the *MAL2* promoter, YPM (1% yeast extract, 2% peptone, and 2% maltose) was used (78). All reagents were purchased from Sigma-Aldrich (St. Louis, MO, USA) unless indicated otherwise.

### Strain construction.

The *rap1*Δ/Δ mutant and the *RAP1*-reintegrated strains were generated using the *SAT1*-flipper method (78). The primers used are listed in Table S2 (in the supplemental material). The 5′ and 3′ flanking regions of *RAP1* were amplified from the SC5314 genome using the primer pair Rap1-UR-F-ApaI and Rap1-UR-R-XhoI and the primer pair Rap1-DR-F-SacII and Rap1-DR-R-SacI, respectively. The resulting 5′ and 3′ flanking regions of *RAP1* were independently cloned into the pSFS2A vector to generate pSFS2AdRAP1 (78). The DNA fragment carrying the regions flanking *RAP1* and the *SAT1*-flipper cassette was excised from pSFS2AdRAP1 via ApaI/SacI digestion. The linear DNA was purified, transformed, and integrated into the C. albicans chromosome between the 5′ and 3′ flanking sequences of *RAP1* via homologous recombination. The nourseothricin-resistant transformants were selected for validation by PCR (79). Then, the cells were grown in YPM to induce *MAL2* promoter-regulated recombinase for *SAT1*-flipper excision from the *RAP1* locus. The heterozygous *RAP1* deletion mutants (*rap1*Δ/*RAP1*) were used for a second round of deletion cassette integration and excision to knock out the second allele of *RAP1*.

To construct the *RAP1*-reintegrated strains, the DNA fragment comprising the *RAP1* promoter along with the full-length *RAP1* coding sequence was amplified from the SC5314 genome using the primer pair Rap1-UR-F-ApaI and Rap1-DR-R-SacI. This fragment was cloned into pSFS2AdRAP1 upstream of the *SAT1*-flipper cassette to replace the original ApaI-SacI fragment, generating pRAP1R. The DNA fragment carrying the full-length *RAP1* gene, the *SAT1*-flipper cassette, and the 5′ and 3′ flanking regions of *RAP1* was excised from pRAP1R, purified, and transformed into the homozygous *RAP1* deletion mutant (*rap1*Δ/Δ) strains. Nourseothricin selection and pop-out of the *SAT1*-flipper cassette were performed as described previously (79). The strains carrying the integration in the first allele of *RAP1* were used to integrate *RAP1* in the second allele. Finally, the successful strain construction was verified by PCR analysis of genomic DNA, using the primer pair RAP1-F and RAP1-R.

### Cell sedimentation and flocculation assay.

Cells were grown overnight in YPD broth, and ~4.8 × 10^9^ cells were transferred to test tubes and photographed immediately. The sedimentation assays were performed at room temperature, and cell sedimentation was recorded at different time points (80). To examine cell flocculation, 400 μL of cell suspension was transferred to each well of a 24-well flat-bottom plate with slow orbital shaking at room temperature for 30 min. Cells were then examined using bright-field microscopy.

### Susceptibility to cell wall-perturbing agents.

Cell sensitivity to cell wall-perturbing agents was determined using a spot assay. The exponential-phase cells were collected by centrifugation and resuspended in sterile phosphate-buffered saline (PBS). Ten microliters of 10-fold serial dilutions was spotted onto SC agar plates with or without calcofluor white (180 μg/mL), Congo red (20 μg/mL), or tunicamycin (2.5 μg/mL, dissolved in dimethyl sulfoxide [DMSO]; Abcam, Cambridge, UK). Cell viability was recorded after incubation at 30°C for 3 days.

### CSH assay.

The CSH assay was performed as previously described (81) with some modifications. Briefly, cells were collected by centrifugation and resuspended in 3 mL of PBS, and the optical density at 600 nm (OD_600_) was measured (*A*_0_). Then, 200 μL of xylene was mixed with the cell suspension and held at 30°C for 30 min to allow phase separation. The aqueous layer was transferred to a polystyrene tube, and its OD_600_ was measured (*A*_1_). The percentage of CSH was calculated as [(*A*_0_ − *A*_1_)/*A*_0_] × 100.

### Measurement of cell wall carbohydrate content.

To assess the total carbohydrate content of the C. albicans cell wall, a phenol-sulfuric acid method was used as previously described (82) with some modifications. Cells were collected by centrifugation and resuspended in 1 mL Tris-EDTA (TE) buffer (pH 8) containing 0.3 g acid-washed glass beads. Cells were disrupted by vortexing for 30 s and placed on ice for 30 s, and this process was repeated six times. The cell wall pellets were collected by centrifugation and resuspended in 1 mL of TE buffer. Subsequently, 200 μL of the suspension was mixed with 1 mL of sulfuric acid (72% [vol/vol]; Fluka Chemie GmbH, Buchs, Switzerland) and 200 mL of phenol (5% [wt/vol]; J.T. Baker, Phillipsburg, NJ, USA). The mixture was incubated at room temperature for 10 min, followed by further incubation at 37°C for 30 min. Absorbance was measured with an iMark microplate absorbance reader (Bio-Rad, Hercules, CA, USA) at 490 nm, using different concentrations (0 to 200 mg/mL) of d-glucose as standards. To quantitate the content of mannan, glucan, and chitin, the cell wall pellets were obtained as described above, treated with sulfuric acid, boiled at 100°C, and neutralized with saturated Ba(OH)_2_ (82). The sample was analyzed using an HPAEC-PAD and a Dionex ICS-5000 system (Thermo Fisher Scientific, Waltham, MA, USA).

### RNA extraction and RT real-time qPCR.

The exponential phase was used for total RNA extraction and reverse transcription for cDNA synthesis (79). For real-time qPCR, the StepOne Plus real-time PCR system (Applied Biosystems, Waltham, MA, USA) and the primers listed in Table S2 were used. In each 15-μL reaction mixture, 30 ng of cDNA, 300 nM (each) forward and reverse primer, and 7.5 μL of Power SYBR green PCR master mixture (Applied Biosystems) were included. The reactions were performed with 1 cycle at 95°C for 10 min, followed by 40 repeated cycles at 95°C for 15 s and 60°C for 1 min. The *ACT1* transcripts were used as the internal control (83, 84). All experiments were performed in triplicate, with three independent experiments for each strain, and the average threshold cycle (*C_T_*) values were determined. Finally, the relative fold change in gene expression was calculated using the 2^−ΔΔ^*^CT^* method (85).

### Protein extraction and Western blotting.

Cells were collected and resuspended in a lysis buffer (50 mM HEPES [J.T. Baker], 5 mM EDTA [J.T. Baker], 1% Triton X-100 [United States Biological, Salem, MA, USA], 4 μM leupeptin, 0.2 mM phenylmethylsulfonyl fluoride [PMSF], 1 mM Na_3_VO_4_, 1 mM NaF [Fluka], 1 μM pepstatin A, and 1 μg/mL aprotinin). Cells were disrupted by vortexing with acid-washed glass beads, and total proteins were extracted as previously described (86). The total protein concentration was determined using a Bradford protein assay kit (Bio-Rad).

To detect activation of the Mkc1, Cek1, and Hog1 MAP kinases, proteins were resolved using 10% sodium dodecyl sulfate (SDS)-polyacrylamide gel electrophoresis (PAGE) as previously described (86). For Western blotting, anti-phospho-p44/42 MAPK antibody (catalog no. 4370; Cell Signaling Technology, Danvers, MA, USA) was used to detect phospho-Cek1 and phospho-Mkc1. Moreover, phospho-Hog1 and total Hog1 were detected using anti-phospho-p38 MAPK (catalog no. 9215; Cell Signaling Technology) and anti-Hog1 antibodies (catalog no. sc-9079; Santa Cruz Biotechnology, Santa Cruz, CA, USA), respectively; Act1 was detected using anti-β-actin antibody (catalog no. GTX09639; GeneTex, Hsinchu, Taiwan). Horseradish peroxidase (HRP)-conjugated IgG (catalog no. GTX213110-01; GeneTex) was used as the secondary antibody, and HRP was detected by the Western Lightning HRP chemiluminescent substrates (PerkinElmer, Waltham, MA, USA). Finally, the blot images were captured using an ImageQuant LAS 4000 biomolecular imager (GE Healthcare, Chicago, IL, USA).

### Assessment of biofilm formation.

Cells were collected and adjusted to an OD_600_ of ~0.01 with 250 μL of SC broth in each well of a 24-well flat-bottom polystyrene microplate. For the assay of biofilm formation, cells were incubated at 37°C with 5% CO_2_ for 48 h (79). The wells were washed twice with sterile PBS to remove nonadherent cells. The extent of biofilm formation was determined as previously described (79), by measuring reduction of 2,3-bis-(2-methoxy-4-nitro-5-sulfophenyl)-2H-tetrazolium-5-carboxanilide (XTT) at 490 nm as previously described (79).

To examine biofilm structure, 3 × 10^5^ cells were placed on a Thermanox plastic coverslip (catalog no. 174950; Thermo Fisher Scientific) that was kept in each well of a 24-well microplate containing 1 mL of SC medium. After incubation at 37°C with 5% CO_2_ for 48 h, biofilm formed on the coverslip was washed, fixed, dehydrated, and dried as described before (79). The biofilms were examined and photographed using an S-4700 type II scanning electron microscope (SEM) (Hitachi).

### Phagocytosis assay.

The murine macrophage cell line J774A.1 was grown in Dulbecco’s modified Eagle’s medium (DMEM) supplemented with Gibco GlutaMAX, 10% Gibco heat-inactivated fetal bovine serum (FBS), and antibiotics-antimycotic (100 U/mL penicillin, 100 μg/mL streptomycin, and 0.25 μg/mL amphotericin B) at 37°C with 5% CO_2_. All the reagents to culture J774A.1 cells were purchased from Life Technologies (Carlsbad, CA, USA). Additionally, C. albicans cells were stained overnight at 4°C with 50 μg/mL fluorescein isothiocyanate (FITC; dissolved in PBS). For the phagocytosis assay, 2 × 10^5^ J774A.1 cells were seeded on each well of a 6-well plate (AGC Techno Glass, Tokyo, Japan) containing a sterile coverslip with 2 mL of DMEM. The FITC-stained C. albicans cells were then added into each well containing J774A.1 cells at a multiplicity of infection (MOI) of 3 and coincubated at 37°C with 5% CO_2_ for 30 min, followed by washing and formaldehyde fixation. Moreover, nonphagocytosed cells were stained with 5 μg/mL calcofluor white at room temperature for 10 min. The percentage of C. albicans cells engulfed was assessed by analyzing at least 100 macrophages per well, using an Axio Imager A1 fluorescence microscope (Carl Zeiss, Jena, Germany).

### Measurement of cytokine production.

C. albicans cells were collected, washed three times with PBS, and resuspended in DMEM without FBS. Additionally, 3 × 10^6^ J774A.1 cells were seeded in each 60-mm culture dish (Corning Incorporated, Corning, NY, USA) containing 3 mL of DMEM. C. albicans cells were then added to each dish of J774A.1 cells (MOI of 3) and coincubated at 37°C with 5% CO_2_ for 24 h. The supernatant was collected and used to detect and quantify different secreted cytokines with cytokine ELISA kits (Invitrogen, Waltham, MA, USA). For the detection of IL-6, priming of macrophages with 0.5 ng/mL LPS (from Escherichia coli, Sigma-Aldrich) for 24 h was performed before C. albicans infection (87).

### Virulence assay using G. mellonella infection model.

To study the role of Rap1 in C. albicans virulence, G. mellonella larvae (weighting 0.1 to 0.2 g) in the final instar stage were used (88, 89). Larvae were injected with C. albicans cells (5 × 10^5^ cells suspended in PBS), incubated at 37°C, and monitored and recorded daily. Survival curves were plotted using the Kaplan-Meier method.

To assess fungal load, 6 × 10^5^ cells of C. albicans (suspended in PBS) were injected into G. mellonella larvae, incubated at 37°C for 24 h. Twenty microliters of hemolymph was collected from individual larva of each infection group (containing 10 randomly chosen larvae), mixed with 20 mL of double-distilled water (ddH_2_O) to lyse the hemocyte, and plated onto YPD agar. The plates were incubated at 37°C for 3 to 4 days, and numbers of CFU were counted.

### Statistical analysis.

The two-tailed Student *t* test was used to determine significant differences between samples. Statistical significance was indicated with a *P* value of <0.05.
