# Supplementary material for: Associations of Rap1 with Cell Wall Integrity, Biofilm Formation, and Virulence in Candida albicans
Source: Microbiol Spectr. 2022 Nov 23;10(6):e03285-22. doi: 10.1128/spectrum.03285-22 (PMC9769648; doi:10.1128/spectrum.03285-22)
Supplement: Supplemental file 1 — Fig. S1 to S4 and Tables S1 and S2. Download spectrum.03285-22-s0001.pdf, PDF file, 1.8 MB [file spectrum.03285-22-s0001.pdf]

Supplemental Figures

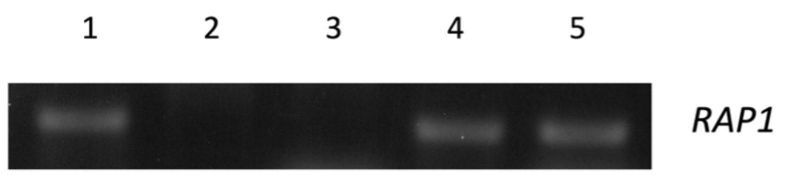

Fig. S1 Validation of *RAP1* gene deletion and reintegration by PCR analysis of genomic DNA.
1: wild type; 2, 3: the *rap1*Δ/Δ mutants; 4, 5: the *RAP1*-reintegrated strains.

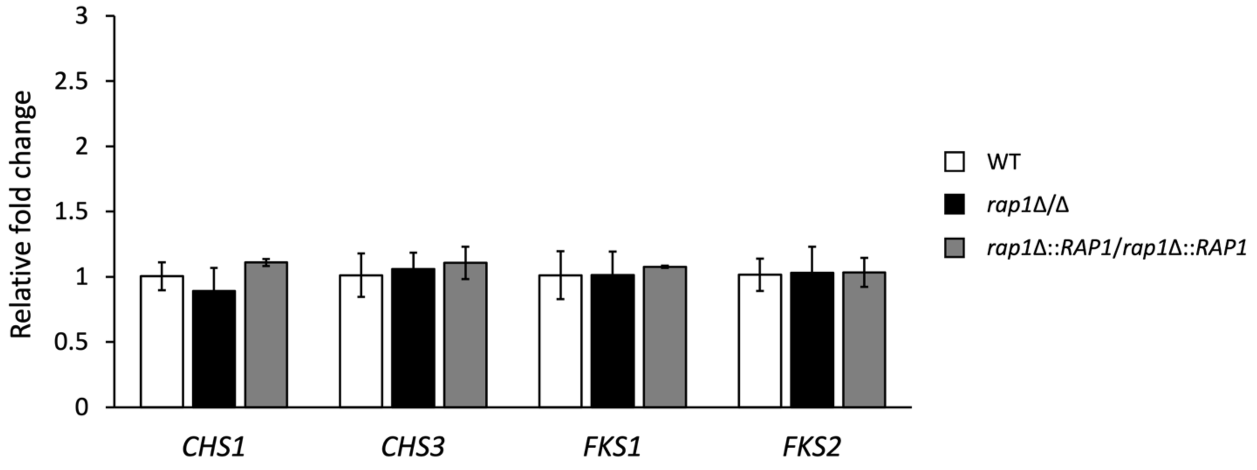

Fig. S2 *RAP1* deletion does not affect the expression of *CHS1*, *CHS3*, *FKS1*, and *FKS2* genes. RT real-time qPCR was performed, and *ACT1* transcripts were used as an internal control. The results are presented as the mean ± SD of at least three independent experiments with three technical repeats in each experiment.

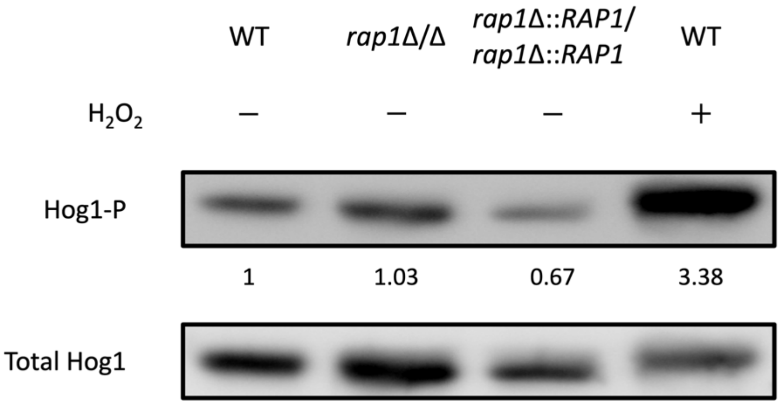

Fig. S3 Hog1 activation is not detected in the *rap1*Δ/Δ mutant. Hog1 activation were assessed by western blotting. Equal amounts of proteins (25 μg) from each sample were loaded. Proteins from cells treated with hydrogen peroxide (H_2O_2) were used as a positive control. The phosphorylated Hog1 (Hog1-P) and total Hog1 were detected by western blotting and analyzed by ImageJ software. Total Hog1 was used as a loading control and was used to normalize Hog1-P level. The phosphorylated level (Hog1-P/total Hog1) was indicated by the fold change levels. The data are representative of three independent experiments with identical results. WT: wild type.

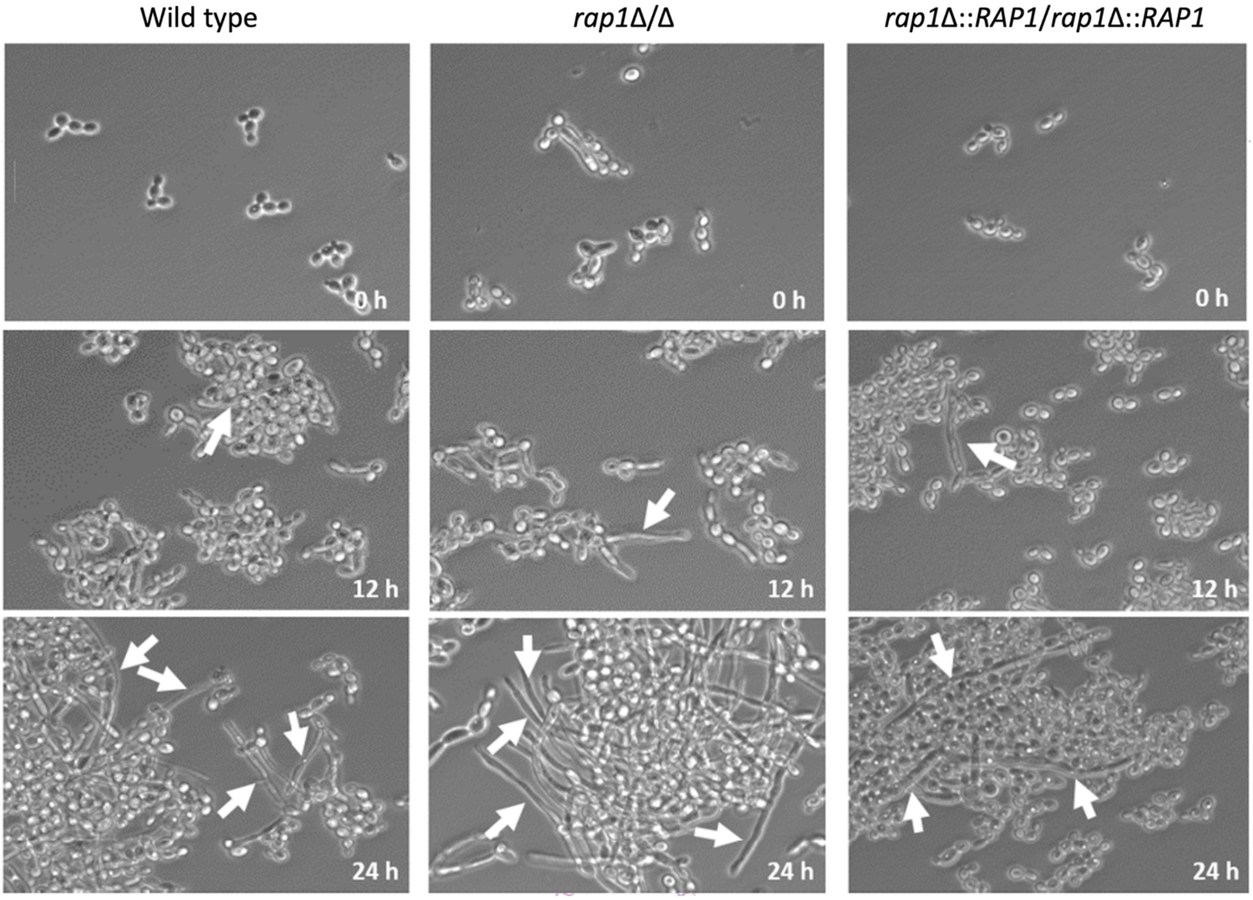

Fig. S4 The *rap1*Δ/Δ mutant exhibits a normal filamentous growth. The filamentous growth of cells was examined in SC medium with 5% CO2 for 12 and 24 h at 37°C. The hyphal cells were examined using a Zeiss microscope, and some representatives were indicated by white arrows.

Supplemental Tables

Table S1 ***C. albicans*** **strains used in this study.**

Strain name	Genotype	Source
SC5314	Wild type	1
The *rap1*Δ/Δ mutant	*rap1*Δ::*FRT*/*rap1*Δ::*FRT*	This study
RAP1-reintegrated strain	*rap1*Δ::*RAP1-FRT*/*rap1*Δ::*RAP1-FRT*	This study

1 Gillum AM, Tsay EY, Kirsch DR. Isolation of the *Candida albicans* gene for orotidine-5'-phosphate decarboxylase by complementation of *S. cerevisiae* ura3 and *E. coli* pyrF mutations. *Mol Gen Genet*. 1984;198(2):179-182.

Table S2 Primers used in this study

Primers	Sequences (5' to 3')
For strain construction[a]	
Rap1-UR-F-ApaI	CGATGGGCCCTGGAGCAGTAAACCGACCAT
Rap1-UR-R-XhoI	ATACCTCGAGTTGTGAAGCCTGAAACCAGA
Rap1-DR-F-SacII	GACTCCGCGGCAATCTTGGAGAGATAGATTTAGGAA
Rap1-DR-R-SacI	CCTAGAGCTCAGCTCGTCCCCATAATTTCA
Rap1-F	TGCAACAAATCAAACCAAATCG
Rap1-R	AAGCATTGATTTCCCATCTTTATCA
For real-time qPCR	
qACT1-F	ATACTCTGTCTGGATTGGTGGTTCT
qACT1-R	TTTTGAAATCCACATTTGTTGGA
qCHS1-F	TTGGAACCGGTGGAACATCT
qCHS1-R	AGACCATAGGTGGACAACAATGAA
qCHS2-F	ATCGACTTGGGGGAAAGATT
qCHS2-R	TGCCAATAATGCTTGTGCTC
qCHS3-F	TGTTTGTTCCTAAAGCTGCTTGTAA
qCHS3-R	AACGACGACGTTGCGATAATAA
qCHS8-F	AAACGAATGCTGATGGATCCA
qCHS8-R	CCAGCGACAATCCATAAAATGA
qFKS1-F	TGATACTGGTAATCATAGACCAAAAA
qFKS1-R	AACTCTGAATGGATTTGTAGAATAAGG
qFKS2-F	ACTTGCTAGCAGTCGCCAAT
qFKS2-R	ACCACCATGAGCGGTTAGAC
qOCH1-F	ATGTCAATCAAATGGGTGCT
qOCH1-R	GGCATACCATCATCTTTCCA
qXOG1-F	GGGTTGGGATGCTAAAAAGGA
qXOG1-R	AATCTGTCAAAGCAGCAGACCAT
qPHR1-F	GCAGTGCTTCAATCAATAGCAAGGC

Table S2 (continued)	
qPHR1-R	AGAGCTTGAGCTGGACCCAGA
qBGL2-F	GTGCCAGTAAACCAGCCATCG
qBGL2-R	CAGTGGAACCTTTAGCAGTTTGA

[a]The restriction sites are underlined.
